# Association among prognostic nutritional index, post-operative infection and prognosis of stage II/III gastric cancer patients following radical gastrectomy

**DOI:** 10.1038/s41430-022-01120-7

**Published:** 2022-03-30

**Authors:** Yanping Xiao, Gang Wei, Min Ma, Dian Liu, Pan Chen, Hu Quan, Jia Luo, Hua Xiao

**Affiliations:** 1 Department of Admissions and Employment, Changsha Health Vocational College, 410010 Changsha, Hunan China; 2 Department of Thyroid and Breast Surgery, Wuhan University Zhongnan Hospital, 430071 Wuhan, Hubei China; 3 Department of Gastrointestinal Surgery, The Third Xiangya Hospital of Central South University, 410013 Changsha, Hunan China; 4 Department of Lamphoma and Abdominal Radiotherapy, Hunan Cancer Hospital and the Affiliated Cancer Hospital of Xiangya School of Medicine, Central South University, 410013 Changsha, Hunan China; 5 Department of Hepatobiliary and Intestinal Surgery, Hunan Cancer Hospital and the Affiliated Cancer Hospital of Xiangya School of Medicine, Central South University, 410013 Changsha, Hunan China; 6 Department of Gastroduodenal and Pancreatic Surgery, Hunan Cancer Hospital and the Affiliated Cancer Hospital of Xiangya School of Medicine, Central South University, 410013 Changsha, Hunan China

**Keywords:** Gastric cancer, Gastric cancer

## Abstract

**Background/objective:**

To investigate the influence of pre-operative immunological and nutritional status, assessed by the prognostic nutritional index (PNI) score, on post-operative infection, and the potential additive effects of low PNI and infection on prognosis after radical resection of stage II/III gastric cancer (GC).

**Methods:**

The medical records of 2352 consecutive stage II/III GC patients who underwent radical gastrectomy were retrospectively reviewed. The independent predictors for infections were identified using univariate and multivariate analyses. Cox regression analysis was used to assess any associations between PNI, infection and OS.

**Results:**

A total of 160 (6.8%) cases developed infections and low PNI (< 43.9) was confirmed as an independent predictor. Both PNI < 43.9 and infections independently predicted poor OS (hazard ratio: 1.163, 95% confidence interval: 1.007–1.343; HR: 1.347, 95%CI: 1.067–1.700), and an additive effect was confirmed as patients with both low PNI and infection had worst OS. Further stratified analyses showed that complete peri-operative adjuvant chemotherapy (PAC, ≥ 6 cycles) could significantly improve OS in patients with low PNI and/or infection, which was comparable to those with PNI ≥ 43.9 and/or infection (*P* = 0.160).

**Conclusions:**

Infection was the most common complication after gastrectomy and PNI < 43.9 was identified as an independent predictor. Low PNI was associated with poorer OS in stage II/III GC, independent of infections, and low PNI and infections had a synergistic effect that was associated with worst OS. However, complete PAC could significantly improve OS in these patients. Thus, strategies to decrease infection and complete PAC should be further investigated.

## Introduction

Gastric cancer (GC) is the fifth most commonly diagnosed malignancy and the fourth leading cause of tumor-induced death worldwide [1]. Unfortunately, more than half of GC patients are diagnosed at the locally advanced stage in China and western countries [2, 3]. To date, surgery offers the only potential curative treatment. With advances of peri-operative treatments and surgical techniques, the incidence of post-operative complications following gastrectomy has markedly declined. But infection still remains a common and sometimes deadly event causing higher hospital expenses and worse survival times [4–8].

The prognostic nutritional index (PNI), which was calculated from the albumin concentration and lymphocyte count, is a frequently used index to reflect the nutritional and immunological status of a patient, has been reported to be related to post-operative complications [9, 10]. Recently, emerging evidence has confirmed that malnutrition and suppressed systemic inflammation, which was assessed by PNI, was confirmed as an independent predictor for a poor prognosis for various types of cancer [9, 11–14]. Given infection was the most common complications and that previous studies have confirmed that malnutrition was adversely associated with post-operative outcomes [3, 9, 15], it seems reasonable to suppose that a low pre-operative PNI could predict infection after gastrectomy. Furthermore, considering that both malnutrition and infection weakens the immune system, and induces a pro-tumor environment [2, 16], an additive impact on prognosis may exist in GC patients having both a malnourished status and infection. However, this hypothesis needs to be rigorously investigated.

Here, we elucidated the impact of low PNI on post-operative infection, and the association among PNI, infection and prognosis of stage II or III GC patients following curative resection, by analyzing data of a high-volume institution in China. Furthermore, we explored whether peri-operative adjuvant chemotherapy (PAC, including both pre-operative and post-operative therapy) could counteract the impairment of a low PNI and infection for long-term outcomes.

## Methods

### Patients and data extraction

A total of 2577 patients with stage II or III gastric adenocarcinoma underwent curative gastrectomy (R0 resection and D2 lymphadenectomy) in the Hunan Cancer Hospital between November 2010 and December 2019. Of these 2577 patients, we excluded 35 with other synchronous cancers, 34 with remnants or recurrence of GC and 156 with acute infection before surgery, missing essential clinical, pathological, or follow-up data; the remaining 2352 patients were included in the subsequent analyses. Each patient signed informed consent for the operation and agreement to use their clinical data prior to surgery. Our retrospective study was authorized by the ethics committee of Hunan Cancer Hospital (No. 27 in 2021).

### Peri-operative management and follow-up

All operations were performed or supervised by highly qualified surgeons. Digestive tract reconstruction and lymphadenectomy were based on the Japanese gastric cancer treatment guidelines and stages in the 8th edition of the TNM classification system [17, 18]. Although radical resection and post-operative adjuvant chemotherapy (AC) was applied to the overwhelming majority of patients with locally advanced GC in our institution, some patients with cT3-4/N + diseases received two to four cycles of neo-adjuvant chemotherapy (NAC) before surgery, using capecitabine/S-1 and oxaliplatin based regimens [17]. Usually, an open procedure was performed in stage II/III GC patients, but a few patients underwent total laparoscopic or laparoscopy-assisted gastrectomy. Prophylactic antibiotics (generally second-generation cephalosporins) were administered to all patients 30 min to 1 h before surgery and continued for 72 h post-operatively.

Post-operative morbidity and mortality within 30 days following surgery were confirmed by the Clavien–Dindo staging system [19]. Due to the lesser clinical importance of grade I complications, the present study analyzed only stage II or greater complications. Fluorouracil and platinum-based adjuvant chemotherapy (AC) was generally started about 28 days following surgery and maintained for about 6 months [20, 21].

Follow-up started 30 days after the operation, and once a quarter in the initial 2 years, every 6 months for the 3rd to 5th years and once a year thereafter. Physical and laboratory measurements were carried out at each follow-up. Ultrasonography or a CT scan was performed every 6 months during the initial 5 years post-operatively and endoscopy every 2 years. The last follow-up time was December 2020.

### Evaluation

Clinicopathological data were collected from medical records and retrospectively analyzed. Laboratory measured were made 7 days prior to surgery, such as routine blood testing and albumin concentrations. The PNI score was calculated as the following equation: [serum albumin (g/L) + 0.005 × total lymphocyte count (/mm^3^)]. The cut-off value of PNI for overall survival (OS) was affirmed by X-tile, as described in previous study [11, 22]. Post-operative infectious complication (such as pulmonary infection, intra-abdominal infection) was diagnosed according to the Centers for Disease Control and Prevention as reported in previous study [3].

OS was defined as the operative day to death of any cause or the end of follow-up. To illustrate the potential addictive influence of low PNI and infection on survivals, patients were divided to four subgroups based on whether they had low PNI or developed infections. Patients who received ≥6 courses of PAC were assigned to the completion group, as patients providing <6 cycles of chemotherapy showed significantly worse prognosis [11, 20].

### Statistical analysis

Data analyses were performed by SPSS software (ver. 24.0, IBM Corporation, New York, US). Measurement data were expressed as the mean ± SD or numbers (%). The difference between groups was investigated by chi-squared test with Fisher’s exact test or Student’s *t*-test (or a Mann–Whitney *U* test), as appropriate. Risk factors for infections were clarified using univariate and multivariate logistic regression analyses. The optimal cutoff PNI score for OS was confirmed using X-tile when reached the maximum χ^2^ log rank value. Survival data were compared by drafting a Kaplan–Meier curve and using the log rank test. Cox proportional hazard regression analysis using a forward conditional method was applied to estimate predictors that may influence OS. All statistical tests were bilateral, and *P*-value <0.05 was considered to be statistically significant.

## Results

### Patients’ Characteristics

Table 1 lists the basic characteristics of the entire 2352 patients. The majority of patients were male (66.5%), performed open procedure (75.0%), for a subtotal gastrectomy (71.9%), with stage III diseases (73.7%), and received PAC (72.2%). Body mass index (BMI) ranged from 13.84 to 37.18 kg/m^2^ with an average of 21.78 kg/m^2^. PNI ranged from 25.70 to 96.10 with an average of 47.47. The mean age, operation time, estimated intra-operative bleeding and post-operative hospital stay was 56.05 years (range, 19–86), 202 min (range, 64–584), 209 mL (range, 50–2300) and 10.9 days (range, 4–144), respectively. Additionally, 258 cases (11.0%) were performed NAC and 543 patients (23.1%) underwent blood transfusion peri-operatively.

**Table 1:** Clinicopathological characteristics of the entire cohort (*n* = 2352).

Variables	PNI < 43.9 group (*n* = 633)	PNI ≥ 43.9 group (*n* = 1719)	*P* value
Gender (males)	437 (60.9%)	1126 (65.5%)	0.107
Age (years)	59.29 ± 10.54	54.85 ± 10.60	0.848
Body Mass Index (kg/m^2^)	20.90 ± 2.84	22.10 ± 3.06	0.010
American Society of Anesthesiology score			<0.001
1	71 (11.2%)	261 (15.2%)	
2	439 (63.4%)	1340 (78.0%)	
3	120 (19.0%)	112 (6.5%)	
4	3 (0.5%)	6 (0.3%)	
Any comorbidities	224 (35.4%)	481 (28.0%)	0.001
Neo-adjuvant chemotherapy	57 (9.0%)	201 (11.7%)	0.064
Pre-operative lymphocyte count (×10^9^/L)	1.31 ± 0.43	1.90 ± 0.67	<0.001
Pre-operative albumin (g/L)	33.45 ± 3.27	40.70 ± 3.52	0.001
Pre-operative hemoglobin (g/L)	101.33 ± 24.5	122.78 ± 23.13	0.002
Operation method			<0.001
Open	534 (84.4%)	1231 (71.6%)	
Laparoscopy	99 (15.6%)	488 (28.4%)	
Type of resection			0.743
Subtotal gastrectomy	458 (72.4%)	1232 (71.7%)	
Total gastrectomy	175 (27.6%)	487 (28.3%)	
Tumor size (cm)	5.12 ± 2.07	4.35 ± 1.94	0.095
Tumor location			0.031
Upper	55 (8.7%)	171 (9.9%)	
Middle	140 (22.1%)	448 (26.1%)	
Lower	406 (64.1%)	1044 (60.7%)	
Diffuse	32 (5.1%)	55 (3.2%)	
pTNM stage*			0.004
II	139 (22.0%)	480 (27.9%)	
III	494 (78.0%)	1239 (72.1%)	
Intra-operative blood loss (mL)	218 ± 153	206 ± 128	0.001
Operation time (min)	202 ± 54	202 ± 55	0.882
Peri-operative blood transfusion			<0.001
Yes	256 (40.4%)	287 (16.7%)	
No	377 (59.6%)	1432 (83.3%)	
Post-operative complications classification^†^			<0.001
None	535 (84.5%)	1562 (90.9%)	
Infectious complications	59 (9.3%)	101 (5.9%)	
Non-infectious complications	39 (6.2%)	56 (3.3%)	
Post-operative complications severity^†^			<0.001
None	535 (84.5%)	1562 (90.9%)	
Grade II	69 (10.9%)	95 (5.5%)	
Grade III or greater	29 (4.9%)	62 (3.6%)	
Requirement of Intensive Care Unit care	22 (3.5%)	44 (2.6%)	0.233
Post-operative hospital stays (days)	11.50 ± 5.21	10.70 ± 5.82	0.838
Peri-operative adjuvant chemotherapy			<0.001
None	229 (36.2%)	424 (24.7%)	
1–5 cycles	271 (42.8%)	741 (43.1%)	
≥6 cycles	133 (21.0%)	554 (32.2%)	

*PNI* prognostic nutritional index.

Data are presented as mean ± SD or *n* (%).

*Tumor stages are based on 8th edition of the Union for International Cancer Control TNM classification.

^†^Defined as Clavien–Dindo grade II or greater. Patients who developed both infectious and non-infectious complications were classified into the infectious group.

The cutoff score of PNI for OS was 43.9 with a maximum χ^2^ log rank value of 19.3017 (Supplementary Fig. 1). Then, a total of 633 patients (26.9%) were diagnosed as poor nutritional and immunological status (PNI < 43.9). Comparison between groups found that a low PNI was associated with lower BMI, lymphocyte count, albumin, and hemoglobin concentrations, higher ASA score, more advanced tumor stage, higher frequency of blood transfusion, but less frequency of PAC (Table 1). Additionally, both post-operative infectious complications and severe complications were more common in patients with a low PNI.

### Post-operative infections

Totally, 255 patients (10.8%) developed 346 adverse events in the initial 30 days after surgery, with 187 (54.0%) infections and 159 (46.0%) cases of non-infections, classified as Clavien–Dindo grade II or higher (Table 2). In particular, 160 patients developed 187 infections, involving 96 cases of intra-abdominal infections ranked first, followed by pneumonia (*n* = 73) and wound infections (*n* = 13). Additionally, 11 patients suffered from infection in those 258 patients (4.3%) who received NAC, which was comparable with those not received NAC (7.1%, *P* = 0.086).

**Table 2:** Post-operative complications of the entire 2352 patients (*n* = 346).

Complications	Number (%)
Infectious	187 (54.0%)
Intra-abdominal infection*	96 (27.7%)
Pneumonia	73 (21.1%)
Wound infection	13 (3.8%)
Sepsis	3 (0.9%)
Urinary tract infection	2 (0.6%)
Non-infectious	159 (46.0%)
Pleural effusion	33 (9.5%)
Ascites	27 (7.8%)
Intestinal obstruction	20 (5.8%)
Gastrointestinal bleeding	20 (5.8%)
Intra-abdominal bleeding	19 (5.5%)
Lymphatic fistula	9 (2.6%)
Cerebral infarction	5 (1.4%)
Anastomotic stricture	3 (0.9%)
Severe arrhythmia	5 (1.4%)
Pneumothorax	3 (0.9%)
Delayed gastric emptying	3 (0.9%)
Urinary retention	3 (0.9%)
Renal failure	3 (0.9%)
Acute attack of chronic obstructive pulmonary disease	2 (0.6%)
Liver failure	2 (0.6%)
Diabetic ketoacidosis	1 (0.3%)
Iatrogenic common bile duct injury	1 (0.3%)

*including those caused by leakage, such as anastomotic leakage, pancreatic fistula, et al.

Univariate analyses found that age ≥ 65 years, ASA score ≥ 3, BMI ≥ 25 kg/m^2^, comorbidity, pre-operative hemoglobin < 100 g/L, operation duration ≥ 240 min, intra-operative bleeding ≥ 300 mL and PNI < 43.9 was possible predictors for infection (*P* value < 0.05, Table 3a). Following multivariate regression analyses including the above mentioned parameters, operation duration ≥ 240 min, PNI < 43.9, comorbidity and BMI ≥ 25 kg/m^2^ were determined as independent predictors (Table 3b).

**Table 3:** (a) Univariate analysis of potential risk factors for post-operative infections following radical gastrectomy for stage II/III gastric cancer (*n* = 2352). (b) Multivariate analysis of potential risk factors for post-operative infections following radical gastrectomy for stage II/III gastric cancer (*n* = 2352).

(a)				
Variables	Infections (*n* = 160)	Non-infection (*n* = 2192)	χ^2^ value	*P* value
Sex (Male: Female)	115/45	1448/744	2.263	0.132
Age (years) ≥ 65/< 65	48/112	499/1693	4.374	0.036
Body mass index (kg/m^2^) ≥ 25/< 25	35/125	303/1889	7.857	0.005
ASA score ≥ 3/<3	27/133	214/1978	8.202	0.004
Comorbidity; yes/no	63/97	642/1550	7.228	0.007
Pre-operative hemoglobin (g/L) ≥ 100/< 100	110/50	1673/519	4.663	0.031
Neo-adjuvant chemotherapy; yes/no	11/149	247/1945	2.947	0.086
Extent of gastric resection; subtotal/total	107/53	1583/609	2.104	0.147
Operation time (min) ≥ 240/< 240*	70/89	475/1716	41.554	<0.001
Intra-operative blood loss (mL) ≥ 300/< 300	50/110	455/1737	9.737	0.002
TNM stage^†^; III/II	119/41	1614/578	0.043	0.837
PNI ≥ 43.9/< 43.9	101/59	1618/574	8.661	0.003

(b)			
Variables	Odds Ratio [OR]	95% Confidence Interval [CI]	*P* value
Operation time ≥ 240 min	2.861	2.050–3.992	<0.001
PNI < 43.9	1.694	1.200–2.392	0.003
Comorbidity	1.557	1.109–2.187	0.011
Body mass index ≥ 25 kg/m^2^	1.607	1.064–2.425	0.024

*ASA* American Society of Anesthesiology, *PNI* Prognostic nutritional index.

*The operation time was missed for one patient in each group.

^†^Tumor stages are based on 8th edition of the Union for International Cancer Control TNM classification.

### Predictors for poorer OS

The follow-up time in this study ranged from 1 to 122 months, with a median of 24 months. The median OS time was 56 months, and the median disease-free survival (DFS) time was 53 months. A total of 304 patients (48.0%) with PNI < 43.9 died during the follow-up time, which was significant more common than those with high PNI (38.0%, *P* < 0.001). Similarly, tumor recurrence occurred in 289 patients with low PNI (45.7%), which was more common compared to those with high PNI (651/1719, 37.9%, *P* = 0.001). Unsurprisingly, patients who received ≥ 6 cycles of PAC had significantly better survival than those receiving 1 to 5 cycles or without PAC (*P* < 0.001, Supplementary Fig. 2).

In the univariate analyses, BMI < 25 kg/m^2^, age ≥ 65 years, ASA score ≥ 3, PNI < 43.9, TNM stage III, blood transfusion, infections and without PAC showed potential relationship with poorer OS (*P* value < 0.05, Table 4). But subsequent multivariate analyses by enrolling the above mentioned variables determined that only stage III (hazard ratio (HR): 3.016, 95% confidence interval (CI): 2.509–3.627, *P* < 0.001), without PAC (HR: 1.610, 95% CI: 1.197–1.855, *P* < 0.001), infection (HR: 1.347, 95% CI: 1.067–1.700, *P* = 0.012), PNI < 43.9 (HR: 1.163, 95% CI: 1.007–1.343, *P* = 0.039) and blood transfusion (HR: 1.170, 95% CI: 1.006–1.361, *P* = 0.042) was significant predictors for poor OS.

**Table 4:** Univariate and multivariate analyses of prognostic factors for overall survival (OS) after radical gastrectomy of stage II/III gastric cancer (*n* = 2352).

Variables	*N*	Median OS (Months)	UV *P* value	MV HR (95% CI)	MV *P* value
Gender					
Male	1563	56	0.975		
Female	789	57			
Age (years)					
≥ 65	547	40	<0.001		0.385
< 65	1805	62			
Body Mass Index (kg/m^2^)					
≥ 25	338	89	0.021		0.100
< 25	2014	53			
ASA score					
≥ 3	241	36	<0.001	1.248 (1.033–1.508)	0.022
< 3	2111	62			
Comorbidities					
Yes	705	63	0.472		
No	1647	55			
Hemoglobin (g/L)					
≥ 100	1783	60	0.179		
< 100	569	48			
Prognostic nutritional index			<0.001		
< 43.9	633	38		1.163 (1.007–1.343)	0.039
≥ 43.9	1719	63			
TNM stage*					
III	1733	36	<0.001	3.016 (2.509–3.627)	<0.001
II	619	NA^†^			
Peri-operative blood transfusion					
Yes	543	34	<0.001	1.170 (1.006–1.361)	0.042
No	1809	63			
Post-operative infectious complication					
Yes	160	29	<0.001	1.347 (1.067–1.700)	0.012
No	2192	60			
Adjuvant chemotherapy					
No	653	34	<0.001	1.610 (1.197–1.855)	<0.001
Yes	1699	68			

*ASA* American Society of Anesthesiologist; *OS* Overall survival; *CI* Confidence interval; *HR* Hazard ratio; *UV* Univariate analysis; *MV* Multivariate analysis.

*Tumor stages are based on 8th edition of AJCC TNM classification.

^†^The median overall survival time has not reached during the follow-up.

### Relationship between low PNI, infections and OS

**Fig. 1:**
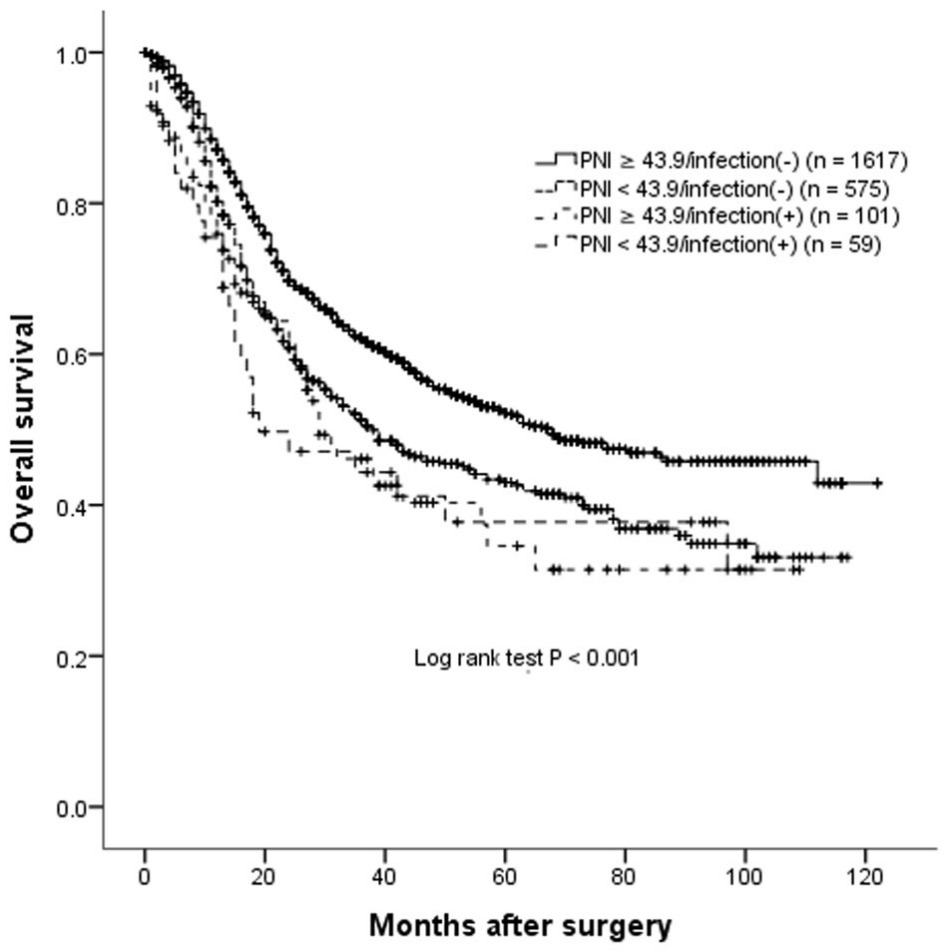
The long-term outcome of the entire cohort patients. Overall survival curves in 2352 patients who underwent radical gastrectomy for stage II/III gastric cancer classified by prognostic nutritional index (PNI) and developing post-operative infection or not. (+) defined as developing post-operative infections.

The median OS in those with higher PNI and not developed infection (the PNI ≥ 43.9/infection (−) group, *n* = 1617) was 68 months, which was significantly longer than 38, 29 and 19 months in the PNI < 43.9/infection (−) (*n* = 575), PNI ≥ 43.9/infection (+) (*n* = 101) and PNI < 43.9/infection (+) group (*n* = 59), respectively. In addition, a synergistic impact was found in the PNI < 43.9/infection (+) group, those patients with even worse prognosis compared to the PNI < 43.9/infection (−) or PNI ≥ 43.9/infection (+) group (*P* < 0.001) (Table 5, Fig. 1).

**Table 5:** Comparison of overall survival (OS) of stage II/III gastric cancer following radical gastrectomy of the entire cohort, stratified by prognostic nutritional index (PNI) and infection (*n* = 2352).

Subgroups	*n* (%)	Median OS (months)	Hazard Ratio (HR)	95% Confidence Interval [CI]	*P* value
PNI ≥ 43.9/infection (−)	1617 (72.3%)	68	Reference	Reference	
PNI < 43.9/infection (−)	575 (20.7%)	38	1.378	1.195–1.590	<0.001
PNI ≥ 43.9/infection (+)	101 (3.7%)	29	1.642	1.237–2.181	0.001
PNI < 43.9/infection (+)	59 (3.3%)	19	1.755	1.200–2.565	0.004

(+) defined as developing post-operative infectious complications.

**Fig. 2:**
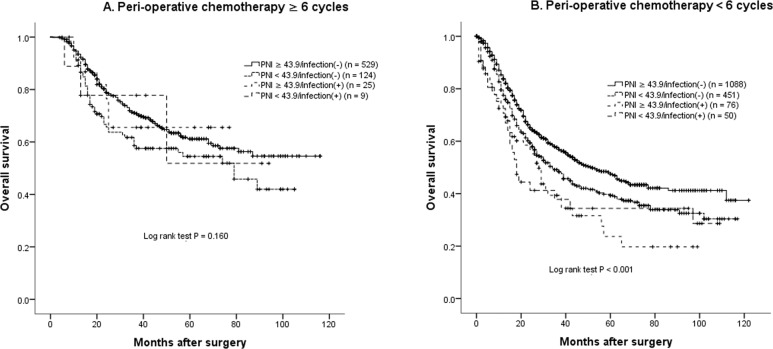
Overall survival curves in 2352 patients who underwent radical gastrectomy for stage II/III gastric cancer classified by prognostic nutritional index (PNI) and developing post-operative infection or not. **A** Patients received complete peri-operative chemotherapy (≥ 6 cycles, *n* = 633); **B** Patients received incomplete peri-operative chemotherapy (< 6 cycles, *n* = 1719). (+) defined as developing post-operative infections.

The median OS for the 687 cases receiving six or more cycles of PAC was not reached in the present study and significantly better than those with less than six cycles of PAC (43 months, *n* = 1665, *P* < 0.001). Further analyses confirmed that the OS time became comparable among the four groups, no matter PNI was high or low, or developing infections (*P* = 0.160, Fig. 2A). But four subgroups of patients with incomplete PAC had significantly different OS (*P* < 0.001, Fig. 2B), similarly as the entire cohort.

## Discussion

This large cohort study demonstrated that not few patients (26.9%) with advanced stage of GC with poor immunological and nutritional status (defined as PNI < 43.9 in the present study), which was in consistence with other previous studies [15, 23]. Possible explanations for relatively higher incidence of malnutrition in locally advanced GC patients included the chronic inflammation, unplanned weight loss related to their cancer process and decreased dietary intake [9, 11, 24]. Serum albumin was the main plasma protein indicating nutritional status, and lymphocyte was the essential component of immune system to eradicate cancer cells. Thus, the PNI could be easily calculated from the albumin concentration and lymphocyte count to represent both the nutritional and immune-inflammatory host status, which might make it a superior index compared to other inflammation-based parameters, such as the neutrophil to lymphocyte ratio (NLR) or Glasgow prognostic score (GPS) [22, 25].

The association between PNI, morbidity and prognosis of GC has recently been investigated [9, 11, 12, 23, 26, 27] but the conclusions remain controversial. Hirahara et al. [12] conducted a retrospective study involving 368 patients who underwent minimally invasive surgery for GC, which revealed that post-operative complications were significantly related to PNI. There was also a multi-center study that reported that the PNI-low group (PNI < 47) experienced a higher incidence of morbidity, but whether low PNI was an independent predictor for complications has not been described [23]. In contrast, Sakurai and colleagues [26] did not find an association between PNI and post-operative morbidity in a cohort of 594 patients treated for GC by gastrectomy. This finding was echoed by Guner and colleagues [27], who argued that a low albumin concentration, instead of PNI, was a risk factor for major complications following curative gastrectomy in 1032 GC patients. The conflicting conclusions between research groups might be due to inconsistent criteria for patient enrollment and the relatively small sample sizes. In the present study, which involved 2352 patients, we demonstrated that a low PNI was a significant risk factor for infection in patients with stage II or III GC who underwent gastrectomy. The relatively larger sample size and the large number of examined variables could offer statistical power to improve the reliability of our conclusions.

With respect to prognosis, a low PNI was found to have an adverse impact on the long-term survival of stage II or III GC patients, a finding in keeping with previous studies [9, 11, 12, 23, 26]. In a meta-analysis conducted by Yang et al. [9], 10 studies involving 3396 GC patients were analyzed. They concluded that a low PNI was correlated with prognosis of stage I to III patients, but not stage IV. Although the exact reason has not been well established, a number of possible explanations have been proposed. First, malnutrition and a low lymphocyte count correlates to immunosuppression and thus provides a favorable micro-environment for tumor recurrence [9, 13]. Second, just as in similar previous studies, we found that low PNI was related to poor patients’ condition (such as a higher ASA score, more common comorbidities) and a more advanced tumor stage (Table 1), which are well-known predictors for prognosis in GC patients that impair survival. Third, as shown in Tables 1 and 3b, low PNI significantly increased the incidence of post-operative infection and was confirmed as an independent risk factor. Further analyses found that both low PNI and infection significantly affected survival and an additive impact was confirmed for the first time, based on the finding that patients who developed an infection and with a low PNI had the worst prognosis. The most likely explanation is that both malnutrition and infection induces inflammation and depresses host immunity, thus accelerating cancer cell proliferation and invasion [6, 10, 11, 13, 17, 28]. As malnutrition has an independent but also an additive effect with infection on long-term outcomes, to decrease infection and improve prognosis, peri-operative immunonutrition supplementation (such as omega-3 fatty acid, arginine, and nucleotide) seems to be a promise therapeutic strategy. Indeed, there is some clinical research, which has shown that immunonutrition reduced the risk of post-operative infectious complications [29, 30]. However, other investigators still failed to demonstrate any clear advantage [31, 32]. In addition, whether nutritional intervention improves survival in malnourished patients has not been fully clarified and needs further investigation.

Last but not least, as shown in Table 1, patients with low PNI seemed less likely to receive or complete peri-operative chemotherapy, as found in our previous study [13]. Our study involved 1288 stage II/III GC patients and PNI < 43.9 was confirmed to be a significant predictor of incomplete PAC. Given that PAC is considered to be the most common and important strategy to decrease tumor recurrence and thus improve prognosis for advanced stage GC after gastrectomy [13, 21], incompleteness of PAC might also partly explain the influence of low PNI on survival times. In the present study, the 5-year OS rate of patients having both low PNI and infection improved from 31.2% to 51.9% if they received six or more cycles of AC, which was comparable to patients with either low PNI or infection, or neither (*P* = 0.160, Fig. 2A). Thus, our conclusions strongly support PAC completion to improve survival in patients with a low PNI and/or infection, a finding echoed by Li and colleagues [33]. In the latter study, which involved a cohort of 206 patients with locally advanced GC who had undergone radical gastrectomy, the patients who suffered from major complications (Clavien–Dindo stage III or greater) were less likely to complete all planned multi-modality therapy. Further analysis found that completion of therapy could compensate for impairment of morbidity on prognosis. Other retrospective studies revealed that performing chemotherapy before surgery could increase the completion of PAC and was a feasible strategy to improve outcomes in patients developing morbidity after gastrectomy [10, 34]. But further prospective research with a larger sample size are still necessary to confirm this hypothesis.

The present study has a number of limitations. First, was its retrospective nature and being single center research. In addition, only Clavien–Dindo stage II or greater complications were analyzed, considering the relatively little clinical importance associated with grade I complications, which inevitably decreased the incidence of infection and thus might impair the reliability of our final conclusions. Second, although a growing number of evidence recommended NAC in patients with locally advanced GC [35], only 258 patients (11.0%) received NAC, and the relationship of response efficiency of NAC and prognosis has not been evaluated in the present study. Moreover, several chemotherapy regimens were used in our institution during the study, such as SOX, XELOX, ECF, FLOT4, et al. [8, 17, 20, 21, 35, 36]. This may also influence the completion of AC and prognosis, as a result, becoming a confounding factor and impair the generalizability of our conclusions. Third, the median follow-up time was only 2 years, which may be too short. However, the overwhelming majority of recurrences should occur within 24 months after resection [37] and in fact 940 patients (40.0%) relapsed during the follow-up period. Notwithstanding these limitations, our research nevertheless has clarified the association among nutrition, infection, and prognosis of stage II or III GC patients following radical gastrectomy, by studying a large cohort of patients.

In conclusion, this study from a high-volume institution in China identified that low PNI could independently predict post-operative infection in stage II/III GC patients who had undergone radical gastrectomy. Both low PNI and infections adversely impacted on survival times, and an additive impact was verified in patients who had low PNI and developed infections. Furthermore, receiving at least six cycles of peri-operative chemotherapy could cancer out the impairment of low PNI and infection on survival times. Thus, strategies to decrease the incidence of post-operative infection and to complete the planned chemotherapy is essential for stage II/III GC patients with low PNI to improve their survival times. Peri-operative immunonutrition supplementation and NAC seem to be possible approaches, but further prospective studies are required.

## Supplementary information

Supplementary Figure 1

Supplementary Figure 2

Supplementary Figure legends

## Data Availability

The database used and/or analyzed during the current study is not publicly available (to maintain privacy) but can be available from the corresponding author on reasonable request.
