# Supplementary figures and images for: Association among prognostic nutritional index, post-operative infection and prognosis of stage II/III gastric cancer patients following radical gastrectomy
Source: Eur J Clin Nutr. 2022 Mar 30;76(10):1449–56. doi: 10.1038/s41430-022-01120-7 (PMC9550621; doi:10.1038/s41430-022-01120-7)

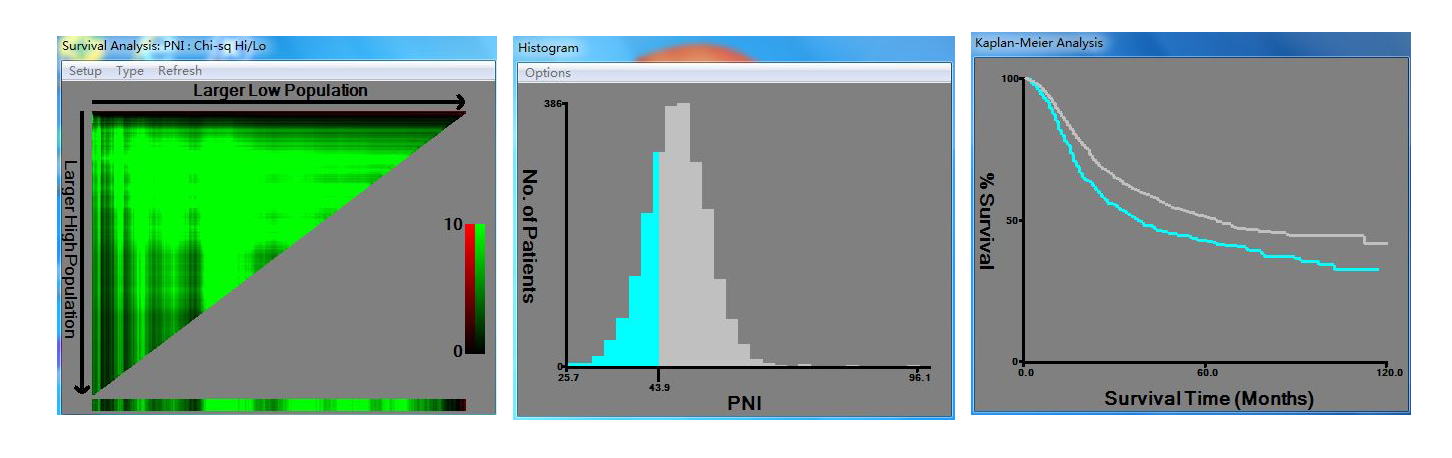

Supplement: Supplementary file 1 — Supplementary Figure 1 [file 41430_2022_1120_MOESM1_ESM.tif]

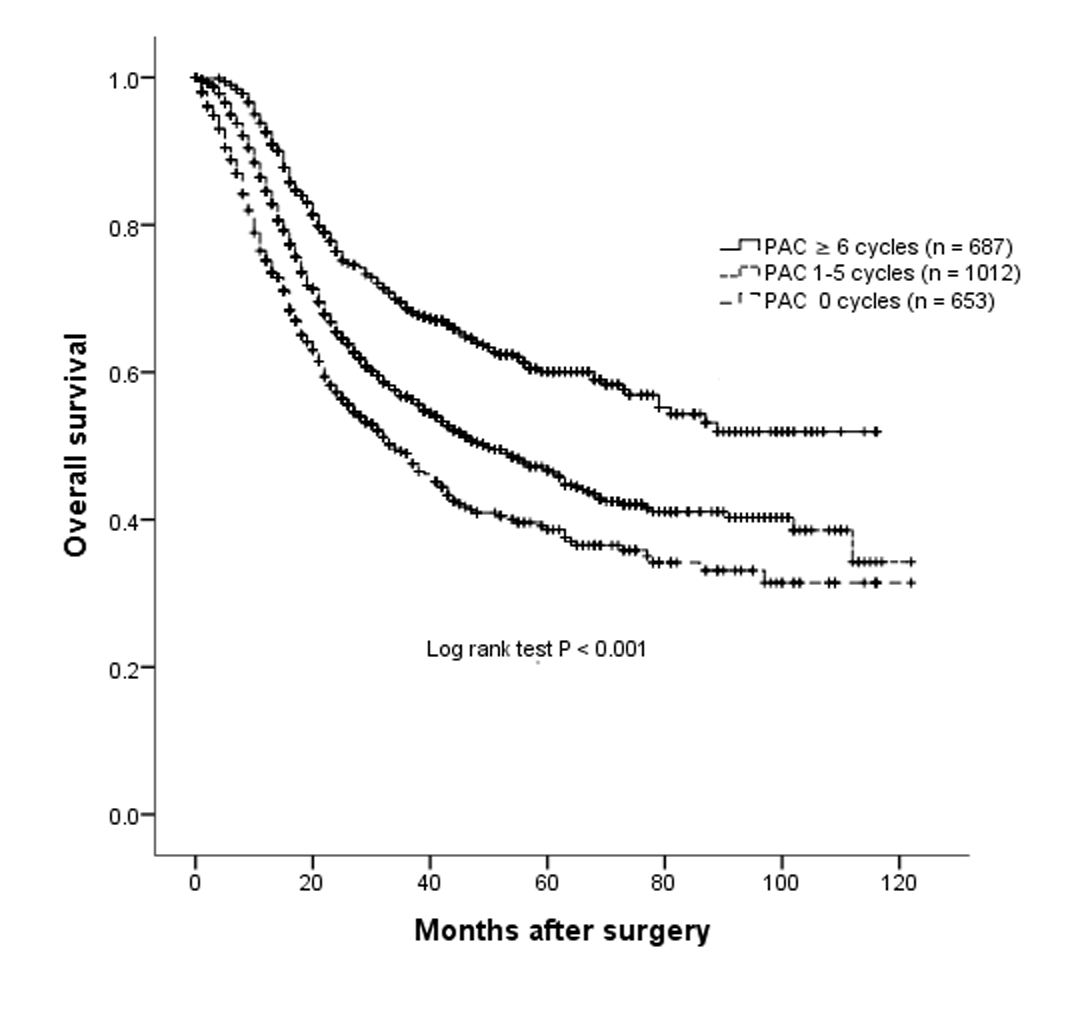

Supplement: Supplementary file 2 — Supplementary Figure 2 [file 41430_2022_1120_MOESM2_ESM.tif]
