# Supplementary material for: Association among prognostic nutritional index, post-operative infection and prognosis of stage II/III gastric cancer patients following radical gastrectomy
Source: Eur J Clin Nutr. 2022 Mar 30;76(10):1449–56. doi: 10.1038/s41430-022-01120-7 (PMC9550621; doi:10.1038/s41430-022-01120-7)
Supplement: Supplementary file 3 — Supplementary Figure legends [file 41430_2022_1120_MOESM3_ESM.doc]

**Supplementary Figure 1.** X-tile analyses of overall survival performed using patients’ data to determine the optimal cut-off values for the prognostic nutritional index (PNI). In the left panels, the X-axis represents all potential cut-off values from low to high (left to right) that define a low subset, whereas the Y-axis represents the cut-off values from high to low (top to bottom) that define a high subset. Red coloration of a cut-off value indicates an inverse correlation with time to recurrence, and the green coloration represents direct associations. The optimal cut-off values highlighted by the black circles in the left panels are shown in the histograms of the entire cohort (middle panels). Kaplan-Meier plots are displayed in the right panels, where blue represents the low subgroup and gray represents the high subgroup. The optimal cut-off values for PNI is 43.9 with a maximum χ^2^ log-rank value of 19.3017.

**Supplementary Figure 2.** Overall survival curves in 2,352 patients who underwent curative resection for stage II/III gastric cancer stratified by the received cycles of peri-operative adjuvant chemotherapy (PAC).
